# Urinary incontinence and sabulous cystitis in mares: report of five cases

**DOI:** 10.29374/2527-2179.bjvm005024

**Published:** 2024-10-21

**Authors:** Ubiratan Pereira de Melo, Cintia Ferreira

**Affiliations:** 1 Veterinarian, DSc. Curso de Medicina Veterinária, Centro Universitário Maurício de Nassau (UNINASSAU). Natal, RN, Brazil.; 2 Veterinarian, MSc. Curso de Medicina Veterinária, UNINASSAU. Natal, RN, Brazil.

**Keywords:** ataxia, equine, lower motor neuron, neuropathy, urinary bladder, ataxia, equino, neurônio motor inferior, neuropatia, bexiga urinária

## Abstract

This report describes five cases of urinary incontinence in Quarter Horse mares associated with the development of sabulous cystitis. The animals in this study had a history of persistent urinary incontinence for eight months, with clinical signs of continuous dribbling urine during rest and movement. The initial treatment with antibiotics and anti-inflammatory drugs was ineffective. Clinical examination revealed severe scalding in the perineal region and on the medial aspect of the hind limbs, along with neurological signs such as ataxia, hind limb paresis, and diminished perineal reflexes. Transrectal ultrasonography confirmed bladder distension and the presence of hyperechoic particles in the urine. Urinalysis revealed typical features of sabulous cystitis, including abundant calcium carbonate crystals. Despite treatment with corticosteroids, antibiotics, and daily bladder lavage with physiological saline, no substantial clinical improvement was observed. The limited therapeutic response and guarded prognosis were discussed with the owner, who opted to monitor the mares without altering their clinical condition for two years. This report highlights the complexity of managing urinary incontinence associated with sabulous cystitis in horses and emphasizes the importance of accurate diagnosis and early recognition of clinical signs for effective treatment.

## Resumo

Este relato descreve cinco casos de incontinência urinária em éguas da raça Quarto de Milha, associada ao desenvolvimento de cistite sabulosa. Os animais deste estudo apresentaram histórico de incontinência urinária persistente por oito meses, com sinais clínicos de gotejamento contínuo de urina, principalmente durante o repouso e o movimento. O tratamento inicial com antibióticos e anti-inflamatórios não foi eficaz. Exames clínicos revelaram escaldaduras severas na região perineal e nas partes internas dos membros posteriores, além de sinais neurológicos como ataxia, paresia dos membros posteriores e reflexos perineais diminuídos. A avaliação por ultrassonografia transretal confirmou a distensão da bexiga e a presença de partículas hiperecóicas no interior da urina. A análise de urina revelou características típicas de cistite sabulosa, incluindo a presença abundante de cristais de carbonato de cálcio. Apesar da administração de tratamento com corticoides e antibioticoterapia, juntamente com lavagem diária da bexiga com solução salina fisiológica, não houve melhora clínica significativa nos animais estudados. A resposta terapêutica limitada e o prognóstico reservado foram discutidos com o proprietário, que optou por manter as éguas em monitoramento sem alteração no quadro clínico por dois anos. Este relato destaca a complexidade no manejo da incontinência urinária associada à cistite sabulosa em equinos, ressaltando a importância de um diagnóstico preciso e do reconhecimento precoce dos sinais clínicos para um tratamento eficaz.

## Introduction

Urinary incontinence (UI), defined as the involuntary voiding of urine, appears to be a relatively uncommon clinical problem in horses. There are several potential causes of UI, which can generally be categorized as non-neurogenic, neurogenic, idiopathic, or myogenic. In horses, non-neurogenic causes include developmental anomalies such as ectopic ureter, urolithiasis, estrogen-responsive incontinence, and incontinence associated with pregnancy and parturition. Neurogenic causes encompass infectious, toxic, traumatic, compressive, degenerative, and inflammatory etiologies (Hines, 2015; Schott, 2006).

UI may indicate primary lower urinary tract disease, neurological disease, or it may be idiopathic. Stretching and inflammation of the bladder wall can have a deleterious effect on the detrusor muscle, leading to a cycle of decreased bladder function and accumulation of crystalloid material (Bayly, 2004).

Diagnosis of incontinence typically involves observing constant or periodic dripping of urine from the vulva or penis. In many cases, bladder dysfunction may persist for months to years before these signs are noticed by owners. Skin scalding, associated alopecia, and the accumulation of calcium carbonate deposits on the hind limbs, perineum (in mares), and ventral abdomen (in males) frequently occur in chronic cases (Mair, 2022).

Sabulous cystitis refers to the accumulation of large amounts of crystalloid urinary sediment in the ventral aspect of the bladder, primarily calcium carbonate crystals (Fonteque et al., 2024; Zakia et al., 2021). Crystalloid material, mainly calcium carbonate, is a normal constituent of equine urine and naturally forms sediment if the bladder does not completely empty during urination. Cystitis often accompanies sabulous accumulation and is presumed to result from constant irritation of the mucosa by crystalloid material and/or ammonia production by bacteria present in the sediment (Rendle et al., 2008).

Historically, the term *sabulous urolithiasis* has been used to describe what is now known as *sabulous cystitis*. Since no actual urolith formation occurs, changing the nomenclature to *sabulous cystitis* has been proposed. The natural process of crystal formation and precipitation that ultimately leads to sediment accumulation in some horses remains unknown. The tendency of suspended particles to settle is governed by forces such as gravity, acceleration, or electromagnetism. The process of sediment formation with particle settling in the bladder can be explained by Stoke’s Law, and it is likely that one or more sedimentation forces act in the horse's bladder (Zakia et al., 2021).

The etiology of sabulous cystitis is uncertain, but it is presumed to represent the final stage of a series of conditions that result in incomplete emptying of the urinary bladder (Bayly, 2004). Since horses normally excrete large amounts of crystalloid material in their urine daily, sabulous cystitis is usually a consequence rather than a cause of urinary incontinence (Schott, 2006).

Current treatment options are limited to repeated bladder lavage, antibiotics, medications promoting bladder emptying, and anti-inflammatory therapy. Bladder dysfunction is usually advanced by the time horses are presented for evaluation due to urine dribbling. The prognosis for horses affected by sabulous cystitis is generally poor due to the persistence or recurrence of clinical signs (Zakia et al., 2021). Urinary incontinence and sabulous cystitis have been linked to a poor prognosis (Holt & Mair, 1990; Keen & Pirie, 2006), particularly as they often coincide with dysfunction of the urethral sphincter and detrusor muscle upon diagnosis (Zakia et al., 2021).

The primary objectives of this retrospective study were to characterize the clinical findings, management, and outcomes of five mares with urinary incontinence and sabulous cystitis.

## Casuistry

Five Quarter Horse mares, used for breeding and aged between 3 and 8 years, presented with a history of urinary incontinence that persisted for eight months. The owner reported that the mares exhibited a staggering gait following an episode of abortion and respiratory disease within the herd, and after 15 d, he observed urine dripping during the daily handling of the mares for reproductive management. Subsequently, the incontinence progressed to continuous dribbling of urine, both at rest and during movement. Normal urination has not been observed since the initial presentation and treatment with antibiotics and anti-inflammatory medications administered by the attending veterinarian failed to resolve the clinical signs.

Mares were fed a commercial concentrate (1 kg/100 kg body weight) and had free access to *Brachiaria* pasture. They received regular deworming every 90 d and were immunized against rabies and tetanus. Two mares were nursing foals of approximately four months old, and two had given birth after the onset of clinical signs. The remaining mare had a history of undiagnosed infertility.

Clinical examination revealed severe urine scalding in the perineal region and on the medial aspect of the hind limbs. Dried urine crystals were evident on the medial aspect of the hind limbs. Neurological examination of all five mares, including assessment of mental status; cranial nerves; muscle tone; spinal reflexes; movement, posture, and postural reactions; revealed ataxia, reduced tone of the external anal sphincter, diminished perineal reflex, hypalgesia of the perineum, and hind limb paresis.

Rectal examination revealed a distended bladder extending cranially into the abdominal cavity, from which urine could easily be expressed using manual pressure. Transrectal ultrasound using a 5 MHz linear probe confirmed bladder distension and revealed hyperechoic particles within the urine, along with a defined hyperechoic line extending from the caudodorsal to the cranioventral region within the bladder.

**Figure 1:**
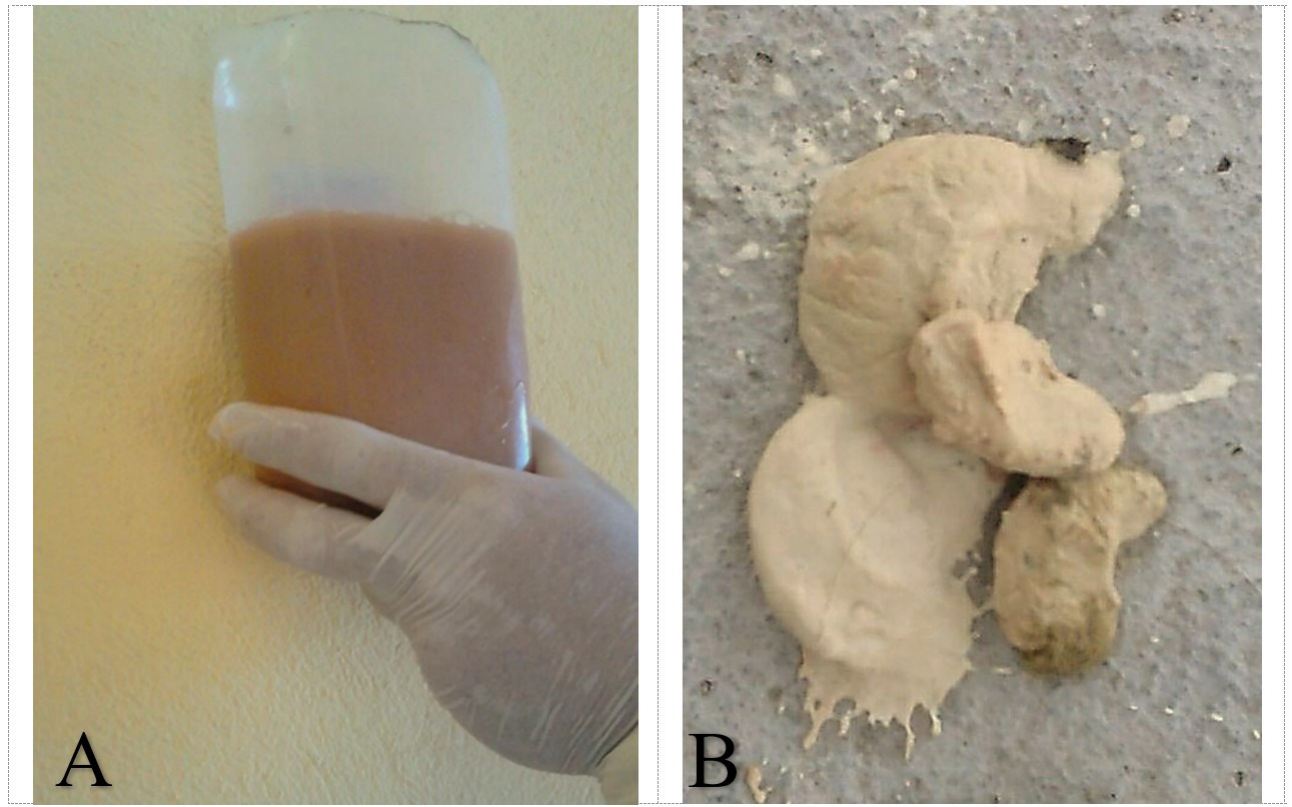
Brownish-colored urine (A) with a sandy appearance, and ‘chalky’ mass (B) eliminated after bladder compression via rectal palpation from five mares with clinical sabulous cystitis.

**Figure 2:**
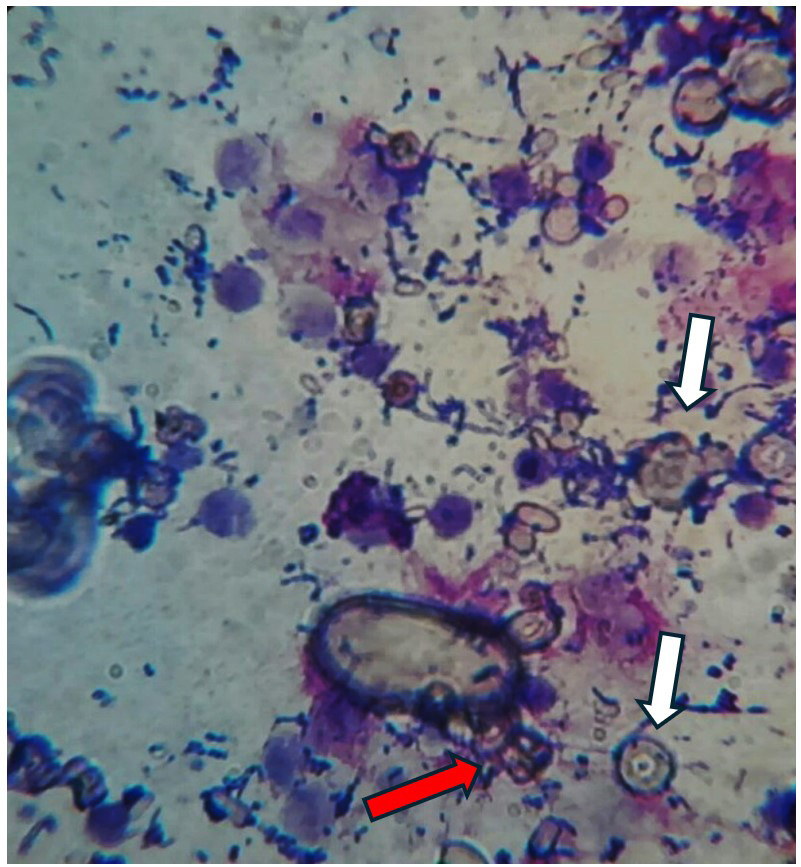
Microscopic analysis of urine, presence of calcium carbonate (white arrows) and calcium phosphate crystal (red arrows) from five mares with sabulous cystitis. 40x magnification. Panotic staining was used.

Urine evacuation was performed using an equine urethral catheter (Figure 1A), and transrectal examination revealed a flaccid and atonic bladder. Additionally, soft masses were palpable on the cranial surface of the bladder (Figure 1B). Hematology and biochemistry (blood urea and creatinine) of the mares showed no abnormalities. Urine samples were collected from the three mares for routine analysis (Figure 2). Urinalysis revealed a brownish-yellow color, foul odor, intensely turbid appearance, and specific densities of 1036 (mare 1), 1044 (mare 2), and 1036 (mare 3). Chemical tests showed proteinuria (+), negative glucose, negative acetone, negative bilirubin, normal urobilinogen, occult blood (++); and pH levels of 8.5 (mare 1), 9.0 (mare 2), and 8.5 (mare 3). Sediment examination indicated rare renal and bladder cells; 1–2 leukocytes per field, 1–3 erythrocytes per field; absence of hyaline and/or granular casts, mucus and rare bacteria; and an abundance of calcium carbonate crystals (+++), amorphous phosphate crystals (++), and calcium phosphate (++). Bacterial culture and antibiotic sensitivity testing were not performed due to lack of owner consent.

The owner was informed of the poor prognosis and limited treatment options. Despite the guarded prognosis of clinical improvement, the owner opted to proceed with the therapy. Initial treatment included Dexamethasone (0.2 mg/kg bwt iv), trimethoprim-potentiated sulphonamide (12.5 g sulphadiazine and 2.5 g trimethoprim b.i.d. PO), and bethanechol chloride (0.03 mg/kg, PO, TID) administered along with bladder lavage using physiological saline solution every other day. However, no clinical improvement was observed following completion of the therapeutic regimen. Treatment with bethanechol chloride was discontinued one month after the mares were examined. The dose was not increased because of the cost, risks of side effects, and the clinician’s impression that it was of little benefit.

Given the lack of response and stable clinical signs, the owner decided to keep the mares in a separate paddock from rest of the herd in the pasture. The mares were monitored for two years without any changes in their clinical condition. During this period, the three mares had one full-term pregnancy.

## Discussion

This report describes five cases of urinary incontinence in mares associated with the development of sabulous cystitis, which is characterized by the accumulation of large quantities of sabulous material within an enlarged, atonic bladder. Although sabulous cystitis is commonly linked to urinary incontinence in horses, there is a paucity of information regarding its pathogenesis in the literature (Keen & Pirie, 2006). An important contributing factor to this lack of understanding is that the affected horses may not be evaluated for months to years after the onset of the clinical problem that eventually leads to sabulous cystitis and urinary incontinence (Schott, 2006; Zakia et al., 2021).

Clinical and laboratory findings were like those reported by Zakia et al. (2021) and Fonteque et al. (2024). Although hematuria was reported as a clinical finding in the case series described by Zakia et al. (2021), in this case series and the case reported by Fonteque et al. (2024), it was not associated with sabulous cystitis, although occult blood was detected during urinalysis. Hematuria appears to be more common in patients with bladder cystoliths (Moraes et al., 2022), and in some cases of leptospirosis (Ferreira et al., 2021).

In the current reported case series, it was not possible to pinpoint the specific cause of the development of urinary incontinence and sabulous cystitis because of the interval between the onset of clinical signs and clinical examination. However, based on the history provided by the owners, the initial cause was presumed to be equine herpes virus type 1 infection. Historically, the neurological causes of incontinence have included equine herpes virus type 1 myeloencephalopathy, equine cauda equina neuritis (equine polyneuritis), intoxication, and sacral trauma. Additionally, a spectrum of neurological diseases accompanied by incontinence may include equine protozoan myeloencephalitis, cervical stenotic myelopathy, and equine degenerative myelopathy (Hines, 2015; Schott, 2006).

Despite the association between urinary incontinence and neurological dysfunction described in the literature (Hines, 2015), Keen and Pirie (2006) and Fonteque et al. (2024) did not report neurological findings in their studies, contrasting with the current report and the findings of Schott et al. (2004) and Zakia et al. (2021). These conflicting findings underscore the importance of conducting a comprehensive and meticulous clinical examination to establish an etiological diagnosis and determine the optimal therapeutic approach for each case.

Traditionally, urinary incontinence has been categorized into two major types: upper motor neuron (UMN) bladder (spastic or reflex bladder) and lower motor neuron (LMN) bladder (paralytic or autonomous bladder). UMN disease is associated with a lesion above the sacral segments in the cord, brainstem, or cortex. The loss of UMN inhibition of the external sphincter typically results in increased urethral resistance, requiring greater intravesicular pressure before voiding occurs. Urinary incontinence associated with UMN dysfunction often initially manifests as intermittent short squirts of urine with incomplete emptying. Although the residual bladder volume varies, the bladder is typically large and turgid, and it is generally difficult to express or catheterize. Over time, chronic bladder distention associated with UMN dysfunction can result in atony and overflow incontinence (Hines, 2015).

A LMN bladder results from a lesion in the sacral segments (S2–4) and associated nerve roots (cauda equina) or peripheral nerves, interrupting the local reflex arc. There is a loss of detrusor function (detrusor areflexia, DA) leading to a paralyzed, atonic bladder. As the bladder becomes overdistended, there is overflow incontinence. The urine dribbling typically appears continuous, which may help distinguish the condition from early UMN bladder dysfunction. The bladder is large and easy to manually express or catheterize. The bladder volume is generally higher than that seen with a UMN bladder. Also, damage to sensory nerve fibers can result in overdistension of the bladder and overflow incontinence, as the transmission of stretch signals from the bladder to initiate the micturition reflex is impaired (Hines, 2015).

Based on the clinical findings identified, urinary incontinence and subsequent sabulous cystitis were due to LMN injury. Disorders typically associated with LMN dysfunction include sacral trauma, equine herpesvirus type 1 myeloencephalitis (EHM), cauda equina neuritis, Sudan grass toxicity, and sorghum cystitis ataxia (Hines, 2015). In this current report, the authors hypothesize that among the cited causes of LMN dysfunction, EHM is the most likely, as it presents with symmetric ataxia and absence of muscular atrophy (Furr, 2015), as observed in this case series. Ferreira et al. (2018) demonstrate the presence of circulating antibodies against EHV-1 in a population of horses kept in the same geographical region where the reported cases occurred, reinforcing the clinical suspicion that the cases were due to EHM.

LMN dysfunction is often accompanied by other signs of sacral or lumbosacral dysfunction, such as loss of external anal sphincter tone, reduced or absent perineal reflex, tail paralysis, analgesia or hypalgesia of the perineum, and paresis of the hind limbs (Mair, 2022), findings identified in all animals in the report.

It is also speculated that horses with lumbar pain that do not adopt a normal urination posture and thus do not completely empty the bladder may be at higher risk of developing sabulous cystitis (Schott, 2006; Zakia et al., 2021). However, in the present report, none of the affected animals had lumbar pain, nor was any predisposing factor for the development of back pain reported. Despite back pain being reported as a predisposing factor for urinary incontinence and sabulous cystitis, a series of reports of horses with back pain examined in the same geographical region as the affected mares did not report urinary incontinence or sabulous cystitis (Melo & Ferreira, 2021).

Schott (2006) cautions that sabulous cystitis can easily be mistaken for a cystolith. However, careful palpation of the bladder mass via rectal examination typically allows for the identification of urinary sediment and differentiation from a cystolith. Moreover, true cystoliths are typically found in small bladders (due to stranguria and pollakiuria), and the concurrent discovery of a large, atonic bladder should prompt the clinician to consider bladder dysfunction and sabulous cystitis.

In the current report, rectal palpation of the five mares revealed the presence of a distended and atonic bladder that was easily emptied upon passage of a urethral catheter. Following bladder evacuation, soft sediment deposits of varying shapes (ellipsoid to oval) were identified in three mares, suggestive of sabulous sediment accumulation. In two mares, these sediments were found at the external urethral orifice.

As discussed by Zakia et al. (2021), bladder lavages and sediment removal have shown to improve clinical signs of urinary incontinence in a case series and may also prevent further damage to the detrusor muscle and delay worsening of the condition. However, complete sediment removal is a time-consuming and challenging process. In their study, bladder lavage under general anesthesia proved effective in two severely affected cases, suggesting this approach could be considered for selected cases. It should be noted, however, that large volumes of physiological saline solution may be required for complete bladder lavage. In contrast to the protocol adopted by Zakia et al. (2021), daily bladder lavages were chosen for this current report.

Consistent with the findings of Schott et al. (2004), who reported unsatisfactory outcomes in the treatment of urinary incontinence in a series of 37 cases, the treatment instituted in this current report also showed inefficacy. Due to the lack of therapeutic response, lifelong clinical management was recommended for the mares to limit urine scalding and ascending infection.

The unfavorable prognosis described in this case series agrees with previous publications (Holt & Mair, 1990; Keen & Pirie, 2006); however, it contrasts with the report by Rendle et al. (2008), which documented successful long-term management of 5 horses with sabulous cystitis. The discordance among different case series may be due to differences in the inciting cause, disease severity, duration, case selection criteria, lack of standardized guidelines for medical management, or different criteria for evaluating clinical response.

The long-term maintenance of horses with urinary incontinence largely depends on the dedication of owners to hygiene and continuous veterinary evaluation and treatment (intermittent bladder lavage and antimicrobial therapy). Additionally, providing a low-calcium diet is recommended to limit urinary sediment accumulation (Schott, 2006).

## Conclusion

This report highlights the complexities involved in the diagnosis and treatment of urinary incontinence associated with sabulous cystitis. The lack of clinical response in many cases and the guarded prognosis emphasize the urgent need for more effective therapeutic strategies and a better understanding of the pathophysiology underlying sabulous cystitis secondary to urinary incontinence in horses. Furthermore, the presence of neurological signs, such as ataxia and hind limb paresis, suggests a possible association with neuropathies, emphasizing the importance of a comprehensive diagnostic approach that includes a detailed neurological evaluation.
